# Hepatic safety of RPV/FTC/TDF single tablet regimen in HIV/HCV-coinfected patients. Preliminary results of the hEPAtic Study

**DOI:** 10.7448/IAS.17.4.19631

**Published:** 2014-11-02

**Authors:** Karin Neukam, Nuria Espinosa, Dolores Merino, Antonio Rivero-Juárez, Ana Carrero, María José Ríos, Josefa Ruiz-Morales, Ana Gómez-Berrocal, Francisco Téllez, Marta Díaz-Menéndez, Antonio Collado, Inés Pérez-Camacho, Marcial Delgado-Fernández, Francisco Vera-Méndez, Juan A Pineda

**Affiliations:** 1 Unit of Infectious Diseases and Microbiology, Hospital Universitario de Valme, Seville, Spain; 2 Service of Infectious Diseases, Hospital Universitario Virgen del Rocío, Seville, Spain; 3 Unit of Infectious Diseases, Complejo Hospitalario de Huelva, Huelva, Spain; 4 Unit of Infectious Diseases, Hospital Universitario Reina Sofía, Instituto de Investigación Biomédica de Córdoba (IMIBIC), Cordoba, Spain; 5 Unit of Infectious Diseases/HIV, Hospital General Universitario Gregorio Marañón, Madrid, Spain; 6 Unit of Infectious Diseases, Hospital Virgen Macarena, Seville, Spain; 7 Unit of Infectious Diseases, Hospital Universitario Virgen de la Victoria, Malaga, Spain; 8 Service of Internal Medicine/Infectious Diseases, Hospital Universitario de la Princesa, Madrid, Spain; 9 CMU of Infectious Diseases and Microbiology, Hospital La Línea, AGS Campo de Gibraltar, La Linea de la Concepcion, Spain; 10 Internal Medicine and Infectious Diseases, Hospital Universitario La Paz/IdiPAZ, Madrid, Spain; 11 Unit of Infectious Diseases, Hospital Torrecárdenas, Almeria, Spain; 12 Unit of Tropical Medicine, Hospital Poniente, El Ejido, Spain; 13 Service of Infectious Diseases, Hospital Regional de Málaga, Malaga, Spain; 14 Section of Infectious Medicine, Hospital Universitario Santa Lucia, Cartagena, Spain; 15 Unit of Infectious Diseases, Hospital Universitario de Valme, Seville, Spain

## Abstract

**Introduction:**

Although hepatotoxicity related to antiretroviral treatment (ART) has become less frequent, hepatotoxic events, such as transaminase elevations (TE), are still a matter of concern. RPV/FTC/TDF (EPA) is a new single tablet regimen which is widely used in real life practice. Clinical trials showed an adequate profile of liver safety in the sub-population of HIV/HCV-coinfected patients receiving rilpivirine. However, the number of individuals included in these analyses is low [1]. The aim of this ongoing study is to evaluate the incidence of TE and total bilirubin elevations (TBE) during the first 48 weeks of EPA-based therapy in a large population of HIV/HCV-coinfected subjects outside of clinical trials.

**Patients and Methods:**

This is a retrospective analysis of HIV/HCV-coinfected subjects who started EPA at the infectious diseases units of 14 centres throughout Spain, included as cases. Subjects who started an ART different to EPA during the study period at the same hospitals were selected as controls. The primary outcome variables were grade 3 or 4 TE and grade 4 TBE.

**Results:**

Of the 191 patients included, 31 (16.2%) subjects were naïve to ART. Eighty-seven individuals started EPA and the remaining ones were controls. The most common NRTI backbone among the controls was TDF/FTC [59 (56.7%) patients] followed by NRTI-sparing regimens [24 (23.1%) individuals] and ABC/3TC [17 (16.3%) subjects]. Among controls, 67 (64.4%) started a ritonavir-boosted protease inhibitor, mainly DRV/r [41 (39.4%) patients] followed by ATV/r [16 (15.4%) subjects]. EFV, ETV and RAL were started in 16 (15.4%), 12 (11.5%) and 13 (12.5%) subjects, respectively. The median (Q1–Q3) follow-up was 5.79 (3.65–8.61) months for the cases and 11.44 (5.8–12.88) months for the controls. TE was observed in two (2.3%) cases versus five (4.8%) controls (p=0.358), accounting for a density of incidence of 4.32/100 person-years versus 5.51/100 person-years [incidence rate difference (95% confidence interval): −1.88 (−9.95–6.2), p=0.354]. All TE were grade 3 and no patient discontinued ART due to TE. None of the cases developed TBE versus four (3.8%) controls, all of them receiving ATV/r.

**Conclusions:**

The frequency of grade 3–4 TE associated with EPA in HIV/HCV-coinfected patients under real life conditions is very low. In addition, TE in HIV/HCV-coinfected patients treated with EPA are usually mild and do not lead to treatment discontinuation. TBE was not seen in patients taking EPA. All these data confirm that EPA is safe in this particular subpopulation.
